# Functional Movement Screen Scores and Physical Performance among Youth Elite Soccer Players

**DOI:** 10.3390/sports5010016

**Published:** 2017-02-21

**Authors:** Bruno Silva, Filipe Manuel Clemente, Miguel Camões, Pedro Bezerra

**Affiliations:** 1 Escola Superior de Desporto e Lazer, Instituto Politécnico de Viana do Castelo, 4960-320 Viana do Castelo, Portugal; filipeclemente@esdl.ipvc.pt (F.M.C.); joaocamoes@esdl.ipvc.pt (M.C.); pbezerra@esdl.ipvc.pt (P.B.); 2 Delegação da Covilhã, Instituto de Telecomunicações, 6201-001 Covilhã, Portugal; 3 Centro de Investigação em Desporto, Saúde e Desenvolvimento Humano, CIDESD, 5000-801 Vila Real, Portugal

**Keywords:** performance, football, FMS, power, sprint, jump, shot speed

## Abstract

This study had two main objectives: (1) to determine if differences in Functional Movement Screen (FMS) scores exist between two levels of competition; and (2) to analyze the association between FMS individual and overall scores and physical performance variables of lower-limb power (jumps), repeated sprint ability and shot speed. Twenty-two Under 16 (U16) and twenty-six Under 19 (U19) national competitive soccer players participated in this study. All participants were evaluated according to anthropometrics, FMS, jump performance, instep kick speed and anaerobic performance. There were no significant differences in the individual FMS scores between competitive levels. There were significant negative correlations between hurdle step (right) and Running-based Anaerobic Sprint Test (RAST) power average (ρ = −0.293; *p* = 0.043) and RAST fatigue index (RAST FatIndex) (ρ = −0.340; *p* = 0.018). The hurdle step (left) had a significant negative correlation to squat jump (SJ) (ρ = −0.369; *p* = 0.012). Rotary stability had a significant negative correlation to RAST fatigue index (Right: ρ = −0.311; *p* = 0.032. Left: ρ = −0.400; *p* = 0.005). The results suggest that individual FMS scores may be better discriminants of performance than FMS total score and established minimal association between FMS scores and physical variables. Based on that, FMS may be suitable for the purposes of determining physical function but not for discriminating physical performance.

## 1. Introduction

Assessment of performance is frequently used to monitor the fitness levels of athletes and the long-term effects of the training process [1]. In the specific case of soccer assessment, batteries include tests to measure the linear speed, change-of-direction speed, aerobic capacity, anaerobic power, lower-body power, isokinetic strength, flexibility, and technical skills [2,3,4]. Despite the different tests used to measure these physical capacities [1], there is a lack of tests that evaluate the movement pattern, commonly assessed by biomechanical techniques [5]. Therefore, the functional movement screen (FMS) has been proposed as a battery test to simplify the assessment of movement patterns in daily sports practice [6].

The specific tests included in the FMS are the deep squat, in-line lunge, hurdle step, shoulder mobility, active straight leg raise, trunk stability push-up, and quadruped rotary stability [7]. The assessment is based on qualitative analysis with a four-point ranking system (from 0 to 3) to evaluate the movement quality [6], since 0 indicates situations in which subjects feel pain and 3 indicates correct performances [8]. A final score from 0 to 21 points summarizes the performance of the participant. Although the FMS can be considered an observational and qualitative method, the interrater reliability scores reveal moderate-to-excellent levels of agreement in trained and untrained raters [9,10]. Therefore, the FMS can be confidently used to assess movement patterns of athletes [10]. 

Some studies have suggested that participants with a final score of 14 and less are exposed to a greater risk of injury [11,12]. Nevertheless, recent studies have suggested that an FMS individual test score can be a better predictor of musculoskeletal injury than the composite FMS score [13,14]. However, FMS battery has been successfully used as a predictor of injury risk [15,16]. Moreover, based on the FMS results, some training programs have been implemented to fix the asymmetries. For example, a study conducted on American football, which investigated the effects of an off-season training program based on movement preparation, found significant improvements in the overall FMS score and asymmetries in comparison with the FMS pre-training program [17]. Another study reported that some movement patterns such as the deep squat, in-line lunge, active straight leg raise, and rotary stability tests improved over a season in collegiate soccer players [18]. 

Some associations between FMS scores and performance variables (strength, power, agility, and speed) have also been analyzed [19,20]. A study conducted in national collegiate golfers did not reveal significant relationships between the FMS and performance variables of 10 m and 20 m sprints, a vertical jump, and an agility T-test [19]. In the specific case of soccer, FMS scores and performance variables were tested in nine female athletes [21] and sprinting tests had no significant correlations with FMS tests. However, unilateral sit-and-reach had positive and significant correlations with the overall FMS score. A 505 change-of-direction speed test also had a positive correlation with the overall FMS test. Finally, a higher-scored left-leg active straight leg raise was related to a poorer unilateral vertical and standing broad jump [21]. The influence of the FMS on performance variables was also tested in the male youth soccer player context [20] and significant correlations were found. Results revealed significant and positive correlations between the FMS score and performance variables of squat jump and reactive strength index, as well as significant and negative correlations with reactive agility. Authors have also reported that in-line lunge explained 47% and 38% of the reactive strength index and reactive agility cut, respectively [20]. In a different study [22], which tested the capacity of the overall FMS score to identify the deficit in speed and jumping performances, the results reported minimal capabilities in such performance variables. 

Despite the preliminary studies conducted in soccer players, results are not conclusive. Moreover, only one study has analyzed the association between the FMS score and performance variables in youth players [20]. More data is needed to analyze the patterns of movement in different youth competitive levels as well as the potential relationships between the FMS and common physical performance variables used in soccer. Therefore, this study had two main objectives: (1) to analyze the variance of the FMS overall score between two youth competitive levels; and (2) to describe the correlations between FMS individual and overall scores and physical performance variables of lower-limb power (jumps), repeated sprint ability and shot speed. Based on previous studies, we hypothesize significant differences at FMS scores between competitive levels and small-to-moderate correlations between FMS individual scores and performance tests. 

## 2. Methods

### 2.1. Participants

Twenty-two Under 16 (age = 15.78 ± 0.52 years; height = 175.00 ± 6.05 cm; body mass = 66.86 ± 4.72 kg) and twenty-six Under 19 (age = 17.32 ± 0.48 years; height = 175.08 ± 6.35 cm; body mass = 69.95 ± 5.53 kg) national competitive soccer players enrolled in a physical evaluation in the Melgaço School of Sports and Leisure biomechanics laboratory. All participants were on the official team by the beginning of preseason, injury-free and passed the federative medical examination. One-on-one interviews with each player were conducted by the main researcher, who was legally responsible for explaining the experimental procedures and observing any apparent physical or psychological diseases. Afterwards, all intervenients signed the Freely-given and Informed Consent Form according to the Declaration of Helsinki. The players were asked to maintain normal daily food and water intake during the study period. All players were familiarized with the experimental procedures and the requirements of the physical and technical tests. Research was approved by the technical–scientific council of the Polytechnic Institute of Viana do Castelo.

### 2.2. Experimental Approach

All participants were evaluated according to anthropometrics, FMS, jump performance, instep kick speed (shot speed) and anaerobic performance. The evaluation took place on two different days, one for each age group. Between 9:30 a.m. and 12:30 p.m., they took part in the laboratory portion, the evaluation of anthropometrics, FMS and jump performance; after 4:00 p.m., in an artificial grass soccer field near the laboratory, the remaining tests were performed. At the beginning and before the FMS assessment, each subject’s age, height, body mass and multi-frequency bioelectrical impedance analysis were recorded. For the field testing, a standardized warm-up was completed, consisting of 10 min of jogging followed by dynamic exercises. After warm-up and before the anaerobic power assessment, the shot speed performance test was accomplished.

### 2.3. Functional Movement Screen

The FMS is a comprehensive screen intended to measure fundamental movement patterns [23], according to seven fundamental movement patterns: the deep squat, hurdle step, in-line lunge, shoulder mobility, active straight leg raise, trunk stability push-up, and rotary stability and three clearing examinations. Three repetitions of each screen were completed, and the best performed repetition was recorded for further analysis [24]. The patterns were evaluated on a scale of 3, 2, 1 and 0, represented according to the relevant criteria: 3—performs the movement correctly without any compensation, complying with standard movement expectations associated with each test; 2—able to complete the movement but must compensate in some way to perform the fundamental movement; 1—unable to complete the movement pattern or is unable to assume the position to perform the movement; 0—pain anywhere in the body [24]. Approximately 10 s of rest were provided between trials, and 1 min between tests. Subjects returned to the starting position between each attempt. After the seven scored patterns, the athlete could achieve a maximum of 21 points. Except for the deep squat and trunk stability push-up, each side of the body was assessed unilaterally. An FMS specialist with three years of experience conducted the tests.

### 2.4. Testing Procedures

#### 2.4.1. Anthropometrics

All participants wore light clothing and stood barefoot, with eyes directed straight ahead. Each subject’s height was measured to the nearest 0.1 cm with a portable stadiometer (SECA 217, Seca Deutschland, Hamburg, Germany). Body composition was analyzed with multi-frequency bioelectrical impedance (Tanita BC-418, Tanita Corp., Tokyo, Japan). This test provided a complete analysis of weight, body mass index, body fat and fat mass percentage, fat-free mass and total body water. Before the assessment, the evaluators manually documented the weight of the clothes, body type, age, and height in the system; the subjects wiped their feet and stood on the weighing platform without bending their knees.

#### 2.4.2. Repeated Ability Sprint test

Anaerobic performance was measured by a Running-based Anaerobic Sprint Test (RAST), a reliable and valid test used to successfully estimate anaerobic capacity and predict short distance performances [25]. 

This test consisted of the performance of six maximal runs (in seconds) of 35 m sprints with 10 s of passive recovery between each sprint. Each effort was timed using two assistants (one at each 35 m spot) and the start for each sprint (10 s interval) occurred with an acoustic sound. In order to carry out a correct and precise testing process, each assistant had a stopwatch and a whistle. Assistant one stood at the start position, and assistant two, at the other line, 35 m apart. The time of the 2nd, 4th and 6th repetitions and the start of the 1st, 3rd and 5th repetitions was the responsibility of assistant one, and assistant two recorded the time of the 1st, 3rd and 5th repetitions and indicated the start of the 2nd, 4th and 6th repetitions. In all repetitions, subjects were encouraged to imprint maximum power and avoid dividing energy between repetitions. The following formula was used to calculate the power (1) for each sprint:

(1) $$Power\;\left(W\right)=\frac{\mathrm{weight}\times {\mathrm{distance}}^{2}}{{\mathrm{time}}^{3}}$$

The fatigue index of the RAST (2) test was determined based on the following formula:

(2) $$\mathrm{Fatigue}\;\mathrm{Index}=\frac{\mathrm{maximum}\;\mathrm{power}-\mathrm{minimum}\;\mathrm{power}}{\mathrm{total}\;\mathrm{time}\;\mathrm{for}\;\mathrm{the}\;6\;\mathrm{sprints}}$$

The higher the fatigue index, the lower the player’s ability to maintain power over the six runs.

#### 2.4.3. Squat and Countermovement Jump

To evaluate the mechanical power of the leg extensor muscles, the vertical jump tests consisted in the Squat Jump (SJ) and Countermovement Jump (CMJ). The assessments were performed on a contact platform (Globus, Italy). Before testing, athletes performed the standard warm-up and were instructed according to the tests’ specifications. Three jump trials were performed with a 15 s rest interval between each trial and 2 min before each test. For the SJ, subjects were instructed to remain in the middle of the contact mat, in a static position, with a 90° knee flexion for 2 s before each jump attempt, without any preparatory movement, and keep their hands on their hips. The procedure for the CMJ is similar, but instead of a static position at 90°, the athlete stands upright and then squats down until the knees are bent at 90° and immediately jumps vertically as high as possible, landing back on the mat. The best result of all the tests was considered for further analysis.

#### 2.4.4. Shot Speed 

Players’ kicking performance was evaluated by the ball’s velocity after an instep kick using a Stalker ATS II Radar System (Applied Concepts, Inc., Plano, TX, USA). The players were instructed to kick, at the fastest speed possible, a stationary ball placed on the penalty point, without a goalkeeper. A standard FIFA-approved ball was used. A radar gun was placed on a tripod and positioned behind the goal, pointing at the ball. The radar gun was calibrated immediately before testing according to the manufacturer’s instructions. The players conducted three trials with the dominant leg and a rest time of 1 min between each attempt. Testing took place in a soccer field with artificial grass. The fastest shot speed (km/h) was selected for the statistical analysis.

### 2.5. Statistical Procedures

Descriptive statistics (mean ± standard deviation (SD)) provided a profile for each parameter. Analysis of variance (ANOVA) of physical variables and total FMS score (scales) were made with the t-independent test followed by the computation of Cohen’s *d* test to measure the effect size (ES). The following scale was used for Cohen’s *d* [26]: 0.41–1.14 (minimum effect); 1.15–2.69 (moderate effect); and >2.70 (strong effect). Analysis of variance between individual FMS scores (ordinal) was made with the Mann–Whitney U test followed by the *r* test to measure the effect size [27]. The following score was used to classify the effect of *r* (ES): small effect is 0.1; medium effect is 0.3; and large effect is 0.5 [27,28]. Spearman correlation was used to test associations between physical variables and individual FMS scores [29]. All statistical analysis was computed using the Statistics Package for Social Sciences (version 23.0; IBM Corporation, New York, NY, USA). 

## 3. Results

Table 1 displays the mean and standard deviation of physical performance between competitive levels. There were significant differences in the weight (*p* = 0.043; *ES* = 0.599, *minimum effect*), SJ (*p* = 0.001; *ES* = 1.241, *moderate effect*), CMJ (*p* = 0.028; *ES* = 0.657, *minimum effect*), shot speed (*p* = 0.031; *ES* = 0.653, *minimum effect*), RAST power average (*p* = 0.021; *ES* = 0.692, *minimum effect*) and RAST fatigue index (*p* = 0.001; *ES* = 1.251, *minimum effect*). U16 players had significantly greater values of SJ, CMJ, RAST power average and RAST fatigue index.

The mean individual FMS scores are shown in Table 2. There were no significant differences in the individual FMS scores between competitive levels. Effect sizes also had no effect in all FMS scores. 

Table 3 displays the correlations between the FMS scores and the SJ, CMJ, Shot Speed, RAST average power, RAST fatigue index and body fat. There were significant and negative correlations of the hurdle step (right) to RAST power average (ρ = −0.293; *p* = 0.043) and RAST fatigue index (ρ = −0.340; *p* = 0.018). The hurdle step (left) had a significant negative correlation to SJ (ρ = −0.369; *p* = 0.012). Rotary stability (right) had a significant negative correlation to RAST fatigue index (ρ = −0.311; *p* = 0.032). There were significant and negative correlations of the rotary stability (left) to RAST fatigue index (ρ = −0.400; *p* = 0.005).

## 4. Discussion 

The results from this study generally supported previous research that established minimal relationships between FMS scores and physical variables. However, U16 players had significantly greater values of SJ, CMJ, RAST power average and RAST fatigue index.

Taking into consideration that speed and explosive power are prerequisites for the success of youth soccer players [30] at the time of this evaluation, U16 athletes had better median indicators of success (Table 1), when compared to U19 counterparts. As we do not control the training loads and the results are related to a specific moment in the season, prudence is required in analyzing the data as a current indicator of performance. Therefore, we are taking into consideration chronological age, when it is more appropriate in these age groups to consider an individual’s maturity status in relation to biological age [31]. Concerning this important age mark, Portas et al. [32], when analyzing FMS scores and maturity status of young soccer players, found that the FMS does not discriminate good and poor movement in U11 players, but is more precise after the mid-youth development phase (U14/U15) when most of the players experienced peak height velocity. This fact and the increased training volume in the older players may mediate the results, as there are no significant differences between FMS scores and competitive levels. However, U19 players had greater average values in five of the seven FMS tests and also a greater average FMS total score.

The mean total FMS score from this study (13.87 ± 2.93 U16 and 14.96 ± 2.07 U19) was different from other studies with similar age groups (15.8 ± 1.8) [33] and soccer players (16.0 ± 2.0) [34]. However, the results are similar to the median scores achieved by Portas et al. [32] when analysed isolated by age groups (U16–13; U19–14). Furthermore, the results supported previous research [19,22,35], as the scoring system generally did not differentiate between athletes with higher or lesser ability levels for speed and jumping. In fact, U16 have superior performance in SJ, CMJ and RAST tests (Table 1) and inferior FMS scores (Table 2).

In soccer practice, the quadricep muscle group plays an important role in jumping and ball kicking and the hamstring controls the running activities and stabilizes the knee during turns or tackles [36]. In another spectrum, the active straight leg raise test assesses active hamstring and gastrosoleus flexibility while maintaining a stable pelvis and core, and active extension of the opposite leg [24]. According to these facts, a significant correlation was expected between FMS tests and lower-limb physical components. In examining the correlations between FMS scores and performance variables (Table 3), there is a clear presence of a statistically significant correlation but with no regular distribution and small-to-medium effect. In fact, and even with this small effect, the significant and negative correlations between rotary stability right (ρ = −0.311; *p* = 0.032) and rotary stability left (ρ = −0.400; *p* = 0.005) with RAST fatigue index may have some indicative implication on running performance. Thus, a lower value in the RAST fatigue index may indicate a greater ability to maintain anaerobic performance, and a higher value in the rotary stability may indicate a better multi-planar trunk stability during a combined upper- and lower-extremity motion [24,37], characteristics of a rapid and explosive running pattern [38]. Considering that the majority of the athletes are right-handed and the RAST test requires a strong dominant start position, this fact may mediate the significant and negative correlation between Hurdle step right and RAST fatigue index (ρ = −0.340; *p* = 0.018).

These indicators. and the fact that our findings are in agreement with other studies reporting weak associations between FMS scores and performance in soccer players [18,20], may lead us to believe that the movement patterns evaluated by the FMS are not important in daily sports practice. However, limitations in mobility, stability, and motor control can cause unnecessary risk in movement health issues, movement competency issues and can also clear the individual for greater investigation into movement capacity [24]. Thus, the FMS must be considered as a screen to individual movement inefficiencies, additional assessment to determine dynamic and functional capacity, and readiness to return to play after rehabilitation from an injury or surgery [24].

Likewise, in soccer, three of the most important perceived risk factors for non-contact injuries are previous injury, fatigue and muscle imbalance, the FMS being the most commonly used screens in others to identify this factor and to predict risk of injury in premier league teams [39].

Considering the limitations of this study, future research should clarify the role of the interaction between FMS and physical performance on injury risk prediction and the role of the improved FMS performance, as an indicator of non-contact injury risk in soccer players. These findings reset the observation of Waldron et al. [40] about the apparent differences between physical performance and physical function, and strongly encourage coaches and practitioners to separately assess each construct.

## 5. Conclusions 

This study found little evidence of association between FMS scores and physical performance in youth soccer players. The results of this study are in line with previous research that established minimal relationships between FMS scores and physical performance. The significant correlations found in our study are relatively weak. In daily soccer practice, the FMS may have an important role in return-to-play strategy, as a screen to access functional mobility and postural stability, helping to manage the preadaptation of the sporting movement as a return-to-play screening tool. The strong intra- and inter-reliability of FMS noted by both novice and expert observers suggests that this screening test can be used in a high range of sports contexts. Based on that, it is suggested that FMS is suitable for the purposes of determining physical function but not for discriminating physical performance.

## Figures and Tables

**Table 1:** Descriptive statistics (mean ± SD) of performance variables and analysis of variance between competitive levels.

Performance Variables	U16 (*n* = 23)	U19 (*n* = 25)	*p*	*Cohen’s d (ES)*
Height (cm)	175.00 ± 6.05	175.08 ± 6.35	0.965	0.013
Weight (kg)	66.86 ± 4.72	69.95 ± 5.53	0.043	0.599
Body fat (%)	15.34 ± 2.16	14.32 ± 1.90	0.134	0.511
SJ (cm)	40.75 ± 5.72	34.43 ± 4.38	0.001	1.241
CMJ (cm)	41.52 ± 5.70	35.50 ± 11.45	0.028	0.657
Shot Speed (km/h)	26.84 ± 1.56	28.04 ± 2.07	0.031	0.653
RAST Power average (W)	575.34 ± 234.76	411.09 ± 239.98	0.021	0.692
RAST Fatigue Index (W/s)	12.35 ± 7.55	5.19 ± 3.24	0.001	1.251
FMS total score	13.87 ± 2.93	14.96 ± 2.07	0.141	0.433

cm: centimeters; kg: kilograms; km/h: kilometers/hour; W: watts; W/s: watts per second; SD: standard deviation; ES: effect size; SJ: squat jump; CMJ: countermovement jump; RAST: Running-based Anaerobic Sprint Test; FMS: functional movement screen.

**Table 2:** Descriptive statistics (mean ± SD) of FMS scores and analysis of variance between competitive levels.

Functional Movement Screen Tests	U16 (*n* = 23)	U19 (*n* = 25)	*Z*	*p*	*r (ES)*
Deep Squat	2.13 ± 0.69	2.08 ± 0.40	−0.430	0.667	0.009
Hurdle Step (right)	1.70 ± 0.47	1.84 ± 0.47	−1.015	0.310	0.021
Hurdle Step (left)	1.57 ± 0.51	1.84 ± 0.47	−1.861	0.063	0.039
In-line Lunge (right)	2.00 ± 0.80	2.32 ± 0.48	−1.470	0.142	0.031
In-line Lunge (left)	2.17 ± 0.58	2.36 ± 0.49	−1.097	0.272	0.023
Shoulder Mobility (right)	2.17 ± 0.89	2.52 ± 0.71	−1.383	0.167	0.029
Shoulder Mobility (left)	2.04 ± 0.93	2.20 ± 0.87	−0.579	0.562	0.012
Straight Leg Raise (right)	2.57 ± 0.66	2.60 ± 0.50	−0.122	0.903	0.003
Straight Leg Raise (left)	2.48 ± 0.59	2.60 ± 0.50	−0.655	0.512	0.014
Trunk Push-up	2.04 ± 0.82	2.20 ± 0.91	−0.847	0.397	0.018
Rotary Stability (right)	1.91 ± 0.42	1.92 ± 0.40	−0.487	0.626	0.010
Rotary Stability (left)	1.83 ± 0.49	2.00 ± 0.00	−1.799	0.072	0.037

**Table 3:** Correlation values (ρ) between physical variables and FMS scores in youth soccer players (*n* = 48).

Physical Variables	SJ (cm)	CMJ (cm)	Shot Speed (km/h)	RAST AV (W)	RAST FatIndex (W/s)	Body Fat (%)
FMS Score Total	−0.124	−0.004	−0.001	−0.142	−0.146	0.05
Deep Squat	0.022	0.079	−0.139	−0.073	0.059	0.111
Hurdle Step (right)	−0.16	0.024	−0.032	−0.293 *	−0.340 *	0.305
Hurdle Step (left)	−0.369 *	−0.281	0.102	−0.035	−0.094	0.129
In-line Lunge (right)	−0.193	−0.036	−0.079	−0.201	−0.183	0.143
In-line Lunge (left)	−0.159	−0.1	−0.171	−0.245	−0.187	0.262
Shoulder Mobility (right)	−0.001	−0.081	0.066	0.057	−0.058	−0.085
Shoulder Mobility (left)	0.076	0.136	0.035	−0.003	−0.009	−0.11
Straight Leg Raise (right)	−0.069	0.025	−0.134	0.152	0.118	0.1
Straight Leg Raise (left)	0.032	0.055	0.032	0.041	−0.038	0.043
Trunk Push-up	0.022	−0.038	0.019	0.043	0.051	−0.258
Rotary Stability (right)	0.053	−0.011	0.12	−0.25	−0.311 *	0.028
Rotary Stability (left)	−0.105	−0.069	−0.061	−0.262	−0.400 **	−0.24

* Correlation is significant at the *p* < 0.05; ** Correlation is significant at the *p* < 0.01. SJ = squat jump; CMJ = countermovement jump; RAST AV = RAST power average; RAST FatIndex = RAST Fatigue index.
